# Genotype-by-sequencing facilitates genetic mapping of a stem rust resistance locus in *Aegilops umbellulata*, a wild relative of cultivated wheat

**DOI:** 10.1186/s12864-016-3370-2

**Published:** 2016-12-15

**Authors:** Erena A. Edae, Pablo D. Olivera, Yue Jin, Jesse A. Poland, Matthew N. Rouse

**Affiliations:** 1 USDA-ARS, Cereal Disease Laboratory, St. Paul, MN 55108 USA; 2 Department of Plant Pathology, University of Minnesota, St. Paul, MN 55108 USA; 3 Wheat Genetics Resource Center, Department of Plant Pathology and Department of Agronomy, Kansas State University, Manhattan, KS 66506 USA

**Keywords:** GBS, High density linkage map, *Aegilops umbellulata*, Wild relatives, Stem rest resistance

## Abstract

**Background:**

Wild relatives of wheat play a significant role in wheat improvement as a source of genetic diversity. Stem rust disease of wheat causes significant yield losses at the global level and stem rust pathogen race TTKSK (Ug99) is virulent to most previously deployed resistance genes. Therefore, the objective of this study was to identify loci conferring resistance to stem rust pathogen races including Ug99 in an *Aegilops umbelluata* bi-parental mapping population using genotype-by-sequencing (GBS) SNP markers.

**Results:**

A bi-parental F_2:3_ population derived from a cross made between stem rust resistant accession PI 298905 and stem rust susceptible accession PI 542369 was used for this study. F_2_ individuals were evaluated with stem rust race TTTTF followed by testing F_2:3_ families with races TTTTF and TTKSK. The segregation pattern of resistance to both stem rust races suggested the presence of one resistance gene. A genetic linkage map, comprised 1,933 SNP markers, was created for all seven chromosomes of *Ae. umbellulata* using GBS. A major stem rust resistance QTL that explained 80% and 52% of the phenotypic variations for TTTTF and TTKSK, respectively, was detected on chromosome 2U of *Ae. umbellulata*.

**Conclusion:**

The novel resistance gene for stem rust identified in this study can be transferred to commercial wheat varieties assisted by the tightly linked markers identified here. These markers identified through our mapping approach can be a useful strategy to identify and track the resistance gene in marker-assisted breeding in wheat.

**Electronic supplementary material:**

The online version of this article (doi:10.1186/s12864-016-3370-2) contains supplementary material, which is available to authorized users.

## Background

Wild relatives of wheat (*Triticum aestivum* L. and *T. turgidum* ssp. *durum* (Desf.)) have been used in breeding programs as sources of agronomically valuable traits. Several genes derived from wild relatives have been deployed in cultivated wheat varieties over the last century and played a significant role in wheat improvement worldwide [1]. Species of the genus *Aegilops* have been successfully used in wheat wide-crossing programs [2, 3]. Though there are many challenges to introgress alien chromatin into wheat, some of the *Aegilops* species have a genome homologous to one of the three *T. aestivum* subgenomes (A, B, and D) and transfer of a favorable trait by conventional crossing is possible [4]. However, introgression barriers for many other species requires the application of techniques such as chemical treatments, irradiation, cold treatment, absence of particular genes such as *Ph1* or gametocidal genes, and bridging crosses [2]. These techniques have facilitated the creation of a range of addition, substitution, and translocation lines through chromatin introgression between wheat and *Aegilops* species such as *Ae. comosa* Sibth & Sm., *Ae. umbellulata* Zhuk., *Ae. geniculata* Roth and *Ae. biuncialis* Vis. [5]. Consequently, numerous studies have demonstrated that *Aegilops* species carry useful genes for traits such as disease and insect resistance [6], drought tolerance [7] and salt tolerance [8]. Resistance genes to leaf rust, stripe rust, stem rust and powdery mildew have been successfully transferred from *Aegilops* species to wheat [9–15].

*Aegilops umbellulata*, a Mediterranean-western Asiatic grass, is one of the 11 diploid species in the *Aegilops* genera [16] that possesses seven pairs of chromosomes (2n = 2x = 14, UU genome). From the standard chromosome karyotype that has been completed for *Ae. umbellulata* [17], six of the seven chromosomes had about the same chromosome size. Relatively, chromosome 1U is the shortest chromosome.

*Aegilops umbellulata* is the source of leaf rust resistance gene *Lr9* that was transferred to hexaploid wheat [18]. This species has also been identified as a source of resistance to stem rust [19, 20], powdery mildew, Hessian fly and greenbug [21]. In addition, it is a source of high-molecular-weight (HMW) glutenin subunits [22, 23].

The search for new sources of resistance to stem rust, caused by *Puccinia graminis* f. sp. *tritici* (*Pgt*), from wild relatives of wheat has been intensified [24] due to the emergence of stem rust pathogen race TTKSK (the first isolate of this race was named Ug99). The cultivated wheat gene pool has a narrow genetic base for resistance to Ug99 and up to 90% of world’s wheat cultivars are considered Ug99 susceptible [25]. Since 2011, a total of five Ug99 resistance genes have been introgressed from *Ae. tauschii* Coss.*, Ae. searsii* Feldman & Kislev ex K. Hammer*,* and *Ae. geniculata* into wheat; these are *Sr51* [26], *Sr53* [27], *SrTA1662* [28], *SrTA10171* [29] and *SrTA10187* [29].

The development of new sequencing technologies has facilitated the discovery of a large number of SNP markers for many crop species such as hexaploid wheat [30], barley [31], rice [32] and maize [33]. Genotyping-By-Sequencing (GBS), a reduced representation genotyping platform, has been used effectively to create high density genetic maps for several cultivated crops such as hexaploid wheat [34, 35], barley [36], oats L. [36] and maize [37]. However, as far as we know, there is no SNP-based genetic map that can be used to map novel traits in *Ae. umbellulata*. Mapping of disease resistance genes in wild diploid progenitor species of wheat has been a successful strategy to aid the cloning of stem rust resistance genes *Sr35* and *Sr22* from *T. monococcum* L [38, 39]. Mapping in a diploid species allows for recombination to readily occur whereas recombination may be inhibited once a genomic region has been introgressed. Only a few U genome-specific markers are available [40, 41]. Therefore, in the present study, we report a new stem rust resistance QTL identified by scanning the entire genome of *Ae. umbellulata* with GBS SNP markers.

## Methods

### Biological materials and genotyping

A total of 140 F_2_ individuals were derived from a cross made between two accessions of *Ae. umbellulata:* PI 298905, resistant to *Pgt* race TTTTF (isolate 01MN84A-1-2) and TTKSK (isolate 04KEN156/04), and PI 542369, susceptible to both races. The two *Ae. umbellulata* accessions were obtained from the publically available National Small Grains Collection (NSGC), USDA-ARS (https://www.ars.usda.gov/pacific-west-area/aberdeen-id/small-grains-and-potato-germplasm-research/docs/national-small-grains-collection/). Leaf tissue was collected from each F_2_ individual and the two parents at the seedling stage, and DNA was extracted following a BioSprint protocol [42]. F_3_ seeds were harvested from each mature F_2_ plant and used for stem rust assays. A single GBS library was constructed for a pool of 142 samples following a GBS protocol with the two restriction enzymes *PstI* (CTGCAG) and *MspI* (CCGG) [26]. Two barcoded adaptors were used for each sample. The two parents were sequenced to a depth 6X the F_2_ individuals. The library was sequenced on the Illumina HiSeq 2000 platform.

### SNP calling and linkage map construction

Raw sequence data were processed for SNPs discovery with the UNEAK algorithm [43] implemented in TASSEL 3.0 [44]. The UNEAK parameters were set as follows: maximum number of expected reads per sequence file 300,000,0000, restriction enzymes used for library construction *PstI-MspI*, minimum number of tags required for output five, maximum tag number in the merged tag counts 200,000,000, option to merge multiple sample per line yes, error tolerance rate 0.03, minimum/maximum minor allele frequencies (MAF) 0.01 and 0.5, and minimum/maximum call rates 0 and 1. The default parameter set was used except for the minimum number of tag counts. SNPs with up to 80% missing data points were retained for subsequent data analysis. However, only SNPs with no missing data, no heterozygous allele states and polymorphic for the two parents were converted into parental genotypes.

SNP data was first converted into parental genotypes for polymorphic SNPs with no missing or heterozygous genotypes for both parents. SNPs with minor allele frequency (MAF) less than 20%, percent heterozygosity greater than 80%, and proportion of missing data points greater than 50% were also removed. Finally, after removing markers with segregation distortion (*p* < 0.01), SNPs with missing data points of less than 10% were used for framework linkage map construction.

Linkage map construction was done in MSTMap software [45] with the following parameters: Distance function Kosambi, cut_off_*p*_value 10^−10^, no_map_dist 15, no_map_size 0, missing_threshold 0.10, estimate_before_clustering no, detect_bad_data yes and objective function ML. Map construction was done four times to stabilize the inflated linkage map size. After each map, double recombinants were changed into missing data guided by graphical genotypes. Markers with missing data points greater than 10% were removed at each step of linkage map reconstruction. Linkage groups from the same chromosome were merged together and reordered.

### Stem rust assay and resistance mapping

The F_2_ individuals and both parents were evaluated against race TTTTF to determine the inheritance of stem rust resistance. F_2:3_ families derived from the same F_2_ plants from which DNA were extracted were assessed for reaction to both races TTTTF (01MN84A-1–2) and TTKSK (04KEN156/04) in the Spring of 2016, under controlled greenhouse conditions, in order to predict the resistance genotype of the corresponding F_2_ plants. Ten to twenty seedlings per family for 124 families and the two parents were assessed for reaction to race TTTTF. A total of 63 families were assessed for reaction to race TTKSK. Experimental procedures for inoculation, incubation, and disease assessment were conducted according to previously described methods [46]. Stem rust seedling infection types were scored based on the 0–4 scale [47] and plants with infection types 0–2 were considered resistant whereas plants with infection types 3–4 were considered susceptible. Each F_2_ plant was classified as homozygous resistant, heterozygous, or homozygous susceptible to each *Pgt* race depending on the segregation of stem rust response of the corresponding F_2:3_ seedlings. Phenotypic segregation pattern of the disease scores recorded on F_2:3_ families were tested for fit to expected segregation ratios using chi-square (*x* ^2^) tests. For the purpose of QTL mapping, F_2_ plants classified as homozygous susceptible, homozygous resistant, or heterozygous were coded as 0, 1, 2, respectively. Quantitative trait loci (QTL) analysis was conducted in R package RQTL with Single QTL mapping (SIM), composite interval mapping (CIM) and multiple QTL mapping (MQM) methods. In addition, we mapped the resistance locus using the assessment of stem rust reaction of F_2_ plants as a molecular marker. The disease score data was included into the genotypic data used to create frame work linkage map using the same linkage map parameters (*see linkage map construction section*).

### Linkage group chromosome assignment

Chromosome assignment of the linkage groups of the framework map was accomplished using draft genome sequences of hexaploid wheat and barley. Chinese Spring Wheat draft genome sequence version 2 [48] and barley whole genome sequence (WGS) [49] were used to anchor the GBS SNP sequences. All 20,252 SNP tags were aligned to the draft sequences using BWA “aln” algorithm with default settings. Then all linkage maps were merged to the output of sequence alignments. Circos tool [50] was used to visualize the syntenic relationship of *Ae. umbellulata* with hexaploid wheat and barley.

## Results

### SNP calling and linkage map construction

From processing raw sequences using *de novo* SNP calling approach in TASSEL 3, a total of 20,252 SNPs were obtained with the default setting except that the minimum tag count was set to five. However, after removing monomorphic SNPs, and also SNPs outside of thresholds set for missing or heterozygous genotypes for any of the two parents, a total of 10,657 SNPs were converted into parental genotypes (A, B and H). After another round of filtering with MAF greater or equal to 20%, level of heterozygosity between 20% and 80%, missing data points up to 10% and segregation distortion *P* < 0.05, a total of 1,933 high-quality SNP markers were selected. These relatively higher quality SNPs were used to construct a framework linkage map using MSTmap software.

Initially, eight linkage groups were obtained from the 1,933 SNPs using MSTMap (Table 1). However, after assigning all linkage groups into chromosomes, two linkage groups were assigned to chromosome 1U, and the remaining six chromosomes had a single linkage group each. The two linkage groups for 1U were merged and the SNPs were reordered. The total linkage map size was 932.47 cM with the average gap size of 0.65 cM (Table 1). We observed chromosome 1U as the shortest linkage group with the largest gap size of 39.96 cM. It also had the least number of markers (47). With the exception of chromosome 3U (max gap 18.75 cM), maximum gap size was less than 7 cM for all of the remaining chromosomes. Chromosome 4U had the largest number of markers (442), and it was also the longest chromosome at 184 cM, followed by chromosome 6U at 172 cM. The chromosome position (cM) and parental genotypes of each SNP in the genetic map is presented in Additional file 1: Table S1.

**Table 1:** Chromosome-wise SNP markers distribution for an *Aegilops umbellulata* biparental population genome-wide map

Chr	#Markers	Map size (cM)	Max. gap size (cM)	Average gap size
1U	47	80.095	39.96	1.74
2U	284	116.82	4.64	0.42
3U	208	118.06	18.75	0.57
4U	442	184.09	4.41	0.42
5U	207	110.74	4.86	0.54
6U	418	172.67	5.75	0.41
7U	327	149.99	6.40	0.46
Total	1,933	932.47		

### Stem rust resistance mapping

**Fig. 1:**
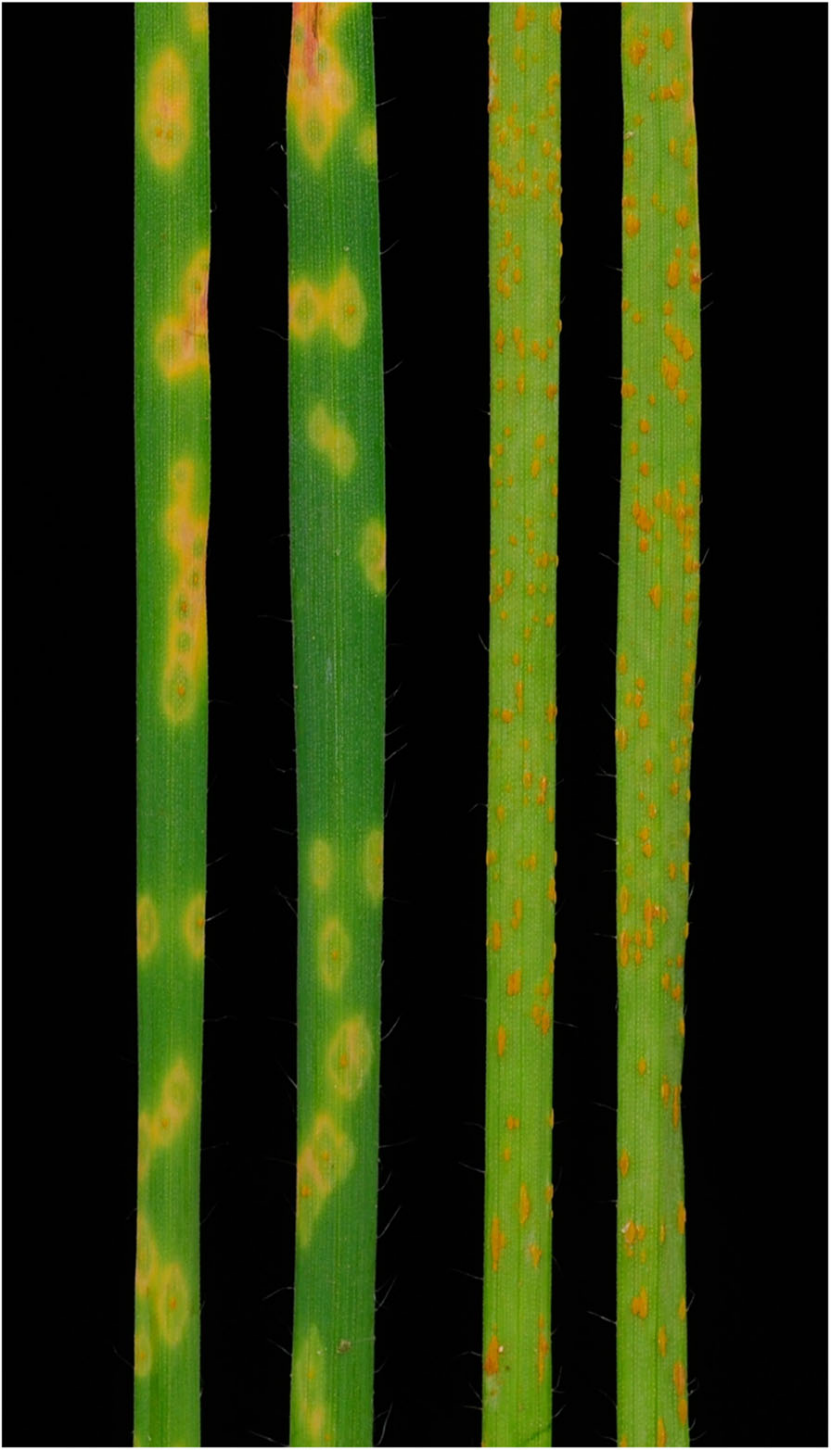
Seedling infection types of PI 298905 and PI 542369 in response to *Puccinia graminis* f. sp. *tritici* race TTKSK. The two leaves on the left are PI 298905 (infection type 2-) whereas the two leaves on the right are PI 542369 (infection type 3+)

Accession PI 298905 was resistant to both *Pgt* races TTTTF and TTKSK with seedling infection type ‘2-’ whereas accession PI 542369 was susceptible to both races with seedling infection type ‘3+’ (Fig. 1). The segregation of disease reaction to races TTTTF and TTKSK significantly deviated from a 1:2:1 ratio for F_2:3_ families (Table 2). However, the segregation pattern across F_2:3_ families was in agreement with a 3:1 ratio for a single dominant gene model (TTTTF, *X* ^2^ = 0.46 *p* = 0.50; TTKSK, *X* ^2^ = 0.42 *p* = 0.52). Taken together, these results suggest that the resistance to each race is conferred by a single gene with dominant effect. In order to test if the gene conferring the resistance to each race is the same, the infection type to races TTTTF and TTKSK were compared. A total of seven F_2:3_ families were homozygous resistant to both races, 32 families were heterozygous to both races, seven families were homozygous susceptible to both races, and one family was susceptible to race TTKSK, but heterozygous to race TTTTF. This co-segregation pattern deviated from the expected ratio for independent resistant loci (*X* ^2^ = 70.3 *p* = 3.90 X 10^−9^). These results suggest that the resistance to races TTKSK and TTTTF resistances is conferred by the same gene or by tightly linked genes at the same locus.

**Table 2:** Number of resistant and susceptible F_2:3_ families of an *Aegilops umbellulata* bi-parental population in response to stem rust pathogen races TTTTF and TTKSK

Race	Homozygous resistant	Heterozygous	Homozygous susceptible	Chi-square(*x* ^2^)	*p*-value
TTTTF^a^	18	69	26	6.59	0.037
TTKSK^a^	9	35	8	6.29	0.04

^a^11 families excluded from TTTTF segregation results and 11 families excluded from TTKSK segregation results due to low samples size of phenotyped F_3_ plants

**Fig. 2:**
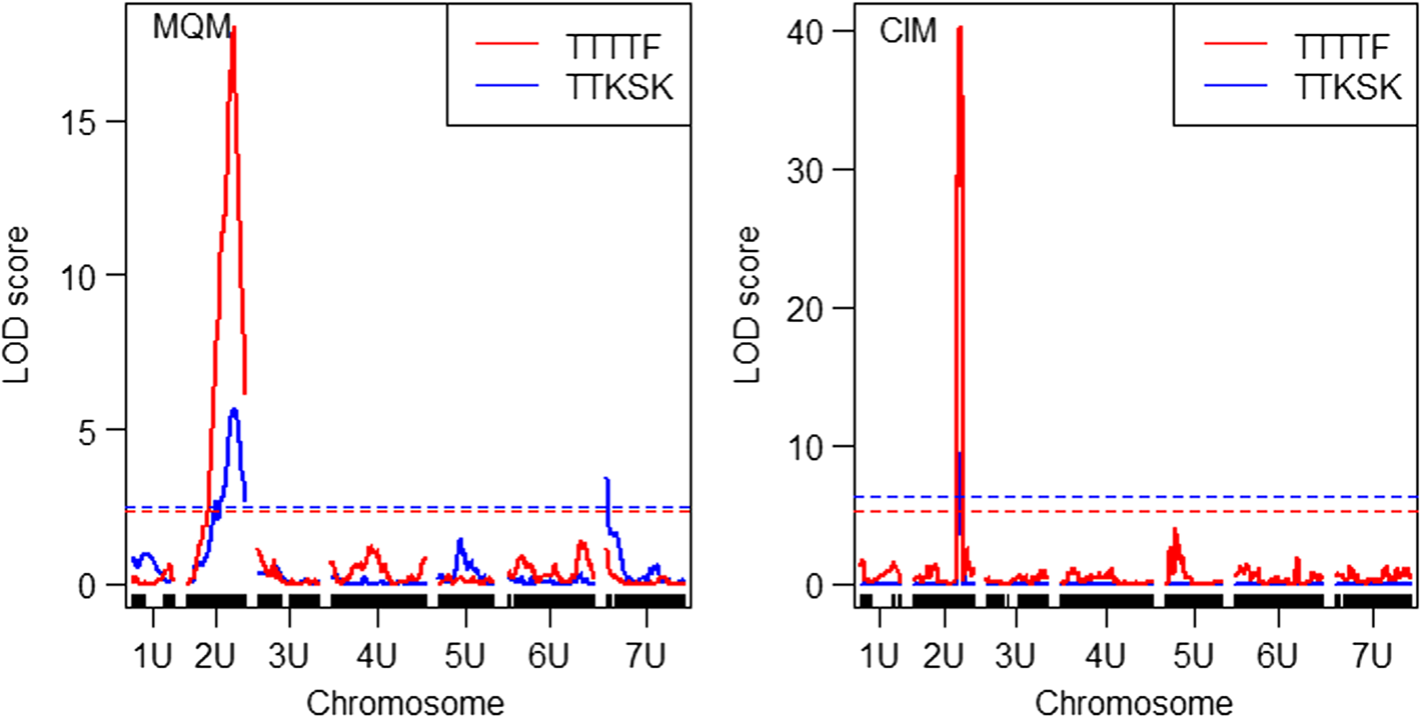
Chromosome and logarithm of odds (LOD) profile of stem resistance QTL detected for races TTTTF and TTKSK in the *Ae. umbellulata* F_2_ population

**Fig. 3:**
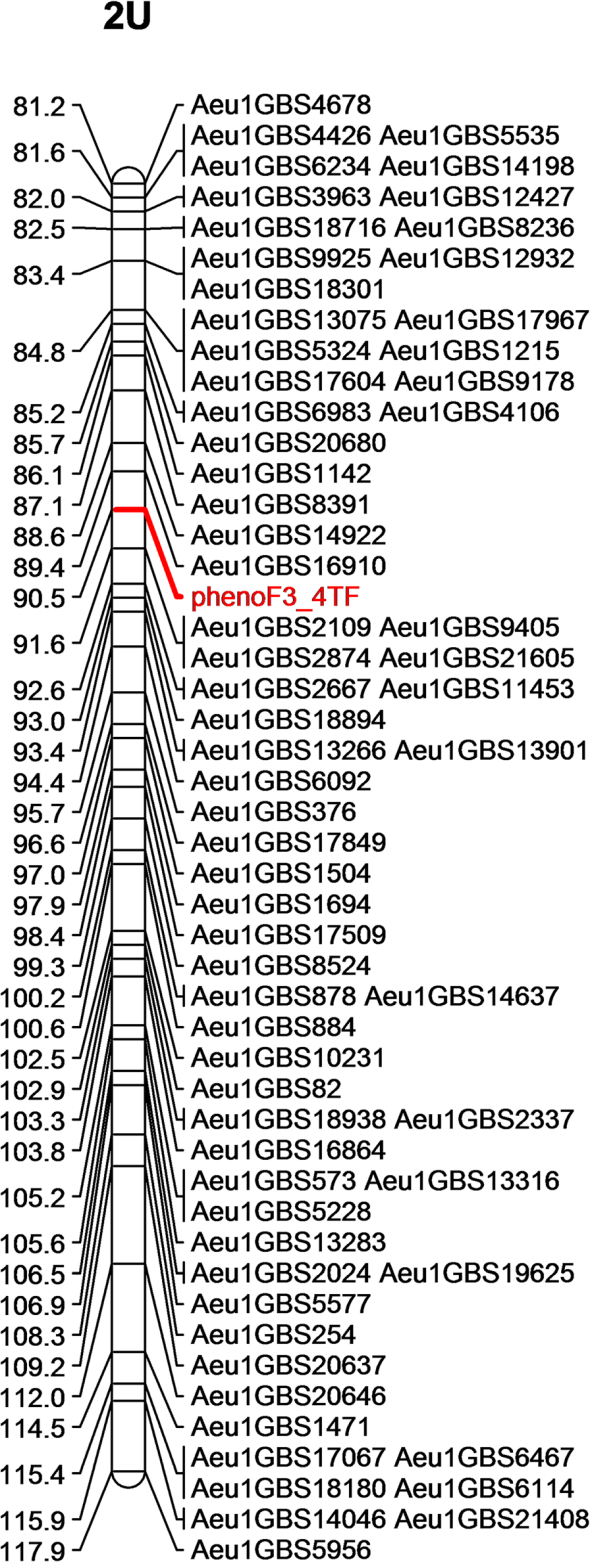
Portion of chromosome 2U showing map location of disease score when mapped as a marker

The disease resistance classification data for races TTTTF and TTKSK of the F_2:3_ families were used for QTL scanning across all seven chromosomes. On the basis of whole-genome scanning using CIM and MQM qtl mapping methods, a major resistance QTL for both races TTTTF and TTKSK was identified on chromosome 2U linked to markers Aeup1GBS11453 and Aeup1GBS13266 (Fig. 2, Table 3). This QTL explained close to 80% of the phenotypic variation for response to race TTTTF and more than 52% of the phenotypic variation for response to race TTKSK (Table 3). Using single qtl mapping methods (SIM) such as Haley-Knot regression (hk), extended Haley-Knot regression (ehk), expectation maximization (em) and non-parametric (np) methods (Additional file 2: Table S2), the QTL associated markers were located within 2–3 cM distance from the major QTL detected on chromosome 2U with multiple QTL methods. A minor QTL was also detected on chromosome 7U for response to race TTKSK (Additional file 2: Table S2, Fig. 2). In addition, the creation of a linkage map with the disease score as a marker placed the phenotype in the QTL region of chromosome 2U between marker Aeup1GBS16910 (89.4 cM) and Aeup1GBS2109/9405 (90.5 cM) (Fig. 3). From sequence similarity search in hexaploid genome sequence databases, best hits were found for marker Aeup1GBS16910 on chromosome 2AL and 2DL; for marker Aeup1GBS2109 on chromosome 2BL and 2DL; for marker Aeup1GBS9405 on chromosome 2DL indicating the QTL detected in this study is on the distal end of the centromere 2U.

**Table 3:** Genotype-by-sequence (GBS) markers linked with quantitative loci (QTL) that confer resistance to stem rust pathogen races TTTTF and TTKSK

Method	Race	Chromosome	GBS Marker	LOD	5% LOD (permutation threshold)	Phenotypic variance (%)	Bayes 95% interval (cM)
CIM	TTTTF	2U@94.2	Aeup1GBS11453	40.3	5.27	79.59	92.2–94.2
	TTKSK	2U@94.2	Aeup1GBS11453	9.49	6.34	59.03	94.2–95.0
MQM	TTTTF	2U@95.0	Aeup1GBS13266	18.03	2.40	78.7	90.0–96.24
	TTKSK	2U@95.0	Aeup1GBS13266	5.65	2.47	52.43	85–105
	TTKSK	7U@0.0	Aeup1GBS16369	3.46	2.47	18.85	0.0–21.02

### Anchoring SNP tags into hexaploid wheat and barley genomes

**Fig. 4:**
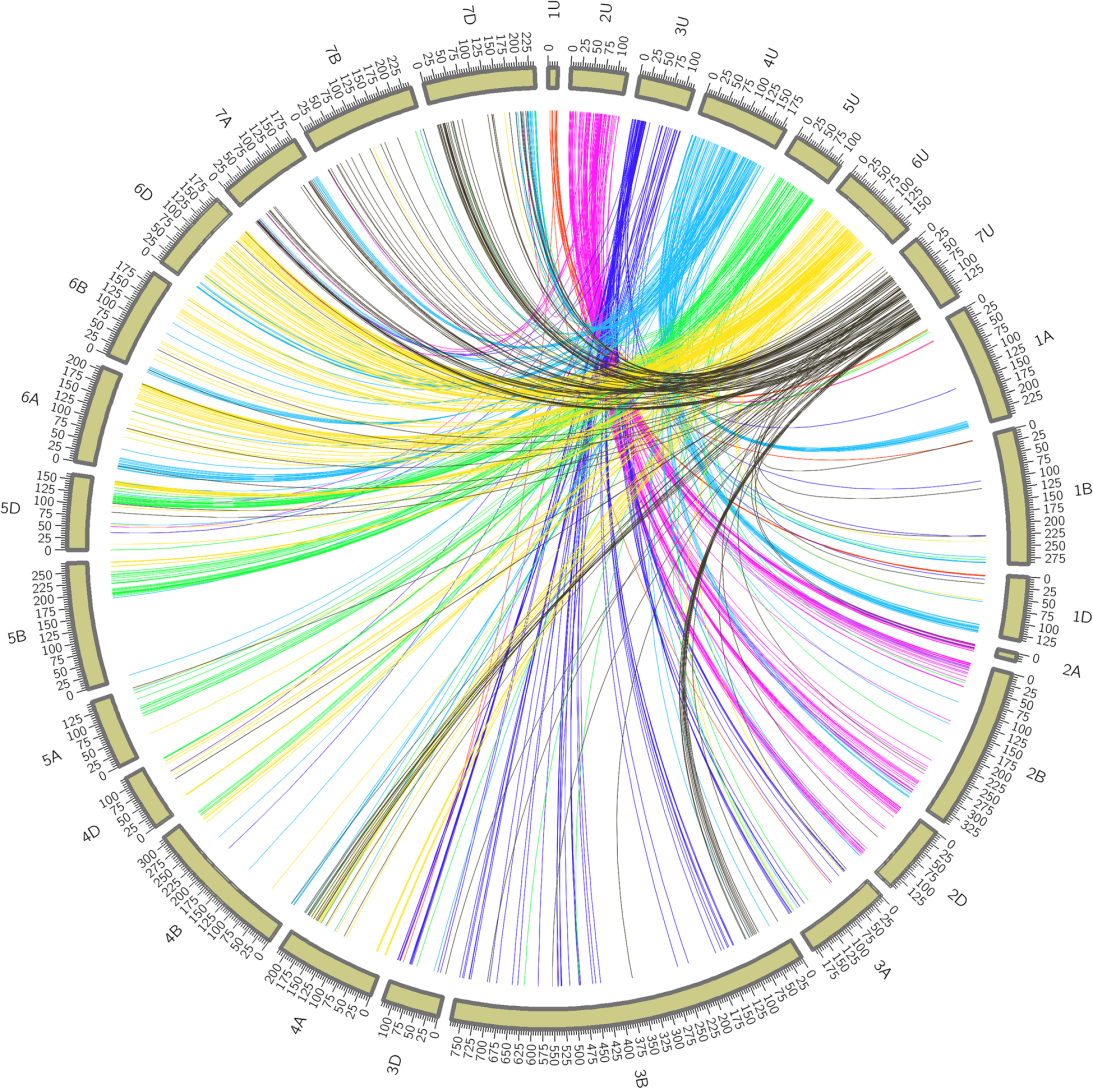
Synthenic relationship between *Ae. umbellulata* and hexaploid wheat using SNP tags of mapped GBS markers

**Fig. 5:**
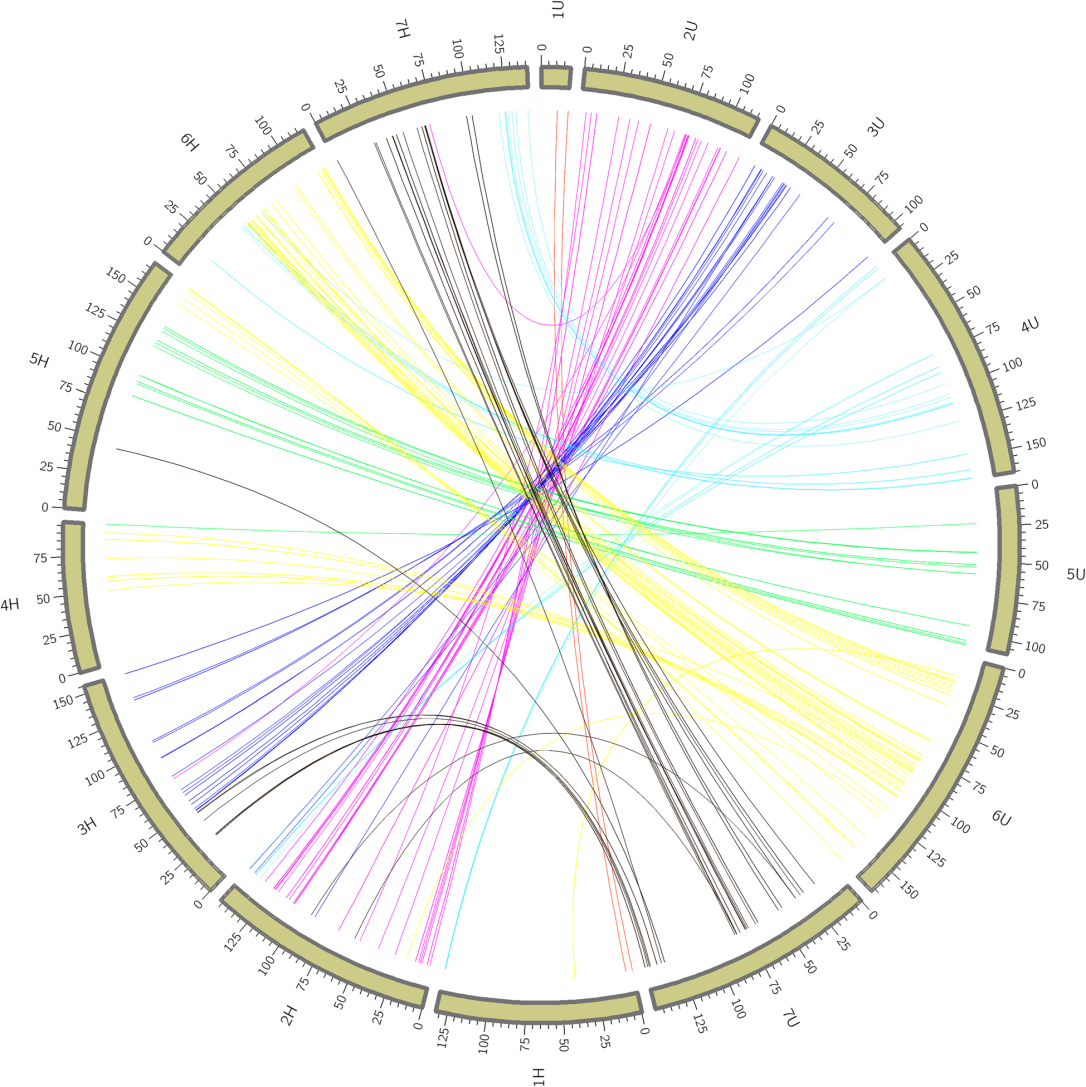
Synthenic relationship between *Ae. umbellulata* and barley (*H. vulgare*) using SNP tags of mapped GBS markers

Uniquely aligned SNP tags using BWA on genome assemblies of hexaploid wheat and barley were used to assign linkage groups to chromosomes. The most hits were found for hexaploid wheat followed by barley. The chromosome similarity of *Ae. umbellulata* with that of hexaploid wheat and barley varied depending on the chromosome. Good collinearity was observed between chromosomes 1U, 2U, 3U and 5U, and group 1, 2, 3 and 5 of hexaploid wheat, respectively (Fig. 4). On the contrary, the segments of the remaining three chromosomes of *Ae. umbelullata* (4U, 6U and 7U) were duplicated across different groups of wheat chromosomes. Segments of chromosome 4U were mainly found on group 1, 6 and 7 of hexaploid wheat whereas chromosome 6U segments were duplicated on chromosomes of group 4, 5, and 6 of hexaploid wheat. Segments of chromosome 7U were found on groups 3, 4 and 7 of hexaploid wheat. For all *Ae. umbellulata* chromosomes, the SNP tags were almost equally distributed across homoleogous chromosomes. A similar syntenic relationship with barley was observed (Fig. 5). With the exception of chromosomes 4U, 6U and 7U, the majority of SNP tags of *Ae. umbellulata* chromosomes were assigned to the corresponding chromosomes of barley. Although the stem rust resistance linked markers on 2U (94–95 cM) did not pass the threshold level used for blast search, other markers in the QTL region such as Aeu1GBS2874 (92.60 cM), Aeu1GBS6092 (96.24 cM) and Aeu1GBS17509 (99.72 cM) showed a match with scaffolds on the long arms of group2 chromosomes of wheat. Furthermore, majority of the markers within 20 cM on the proximal end of the QTL peak were consistently assigned to the long arms of group 2 chromosomes of wheat.

## Discussion

The discovery of novel genes from alien sources and transfer to the domesticated gene pool is an efficient, cost-effective and environment-friendly strategy to combat rust epidemics including stem rust. In this line, many wild relatives of wheat have been used as a source of wheat rust resistance genes in wheat breeding programs [51]. Molecular markers have been found promising for introgression of favorable QTL/genes that confer disease resistance [52]. In the current study, we mapped a novel major stem rust resistance QTL from *Ae. umbellulata*, a diploid wild relative of cultivated wheat, using a bi-parental population genotyped with GBS technology. As there was no previously constructed genetic linkage map for *Ae. umbellulata*, a framework linkage map was created with a total of 1,933 SNPs. The total linkage map size across seven chromosomes of *Ae. umbellulata* was 932.47 cM with the average gap size of 0.65 cM. Although chromosome 1U was the shortest chromosome on the previously reported *Ae. umbellulata* chromosome karyotype [17], the short linkage group size obtained here for chromosome 1U was mainly due to the absence of markers residing in the centromeric region. This resulted in a gap of about 40 cM between the two arms of the chromosome. The poor marker coverage for chromosome 1U could be due to removal of a large number of markers due to segregation distortion, low minor allele frequency and high missing data. Overall, markers were fairly evenly distributed for the remaining six chromosomes. However, complete marker coverage for all chromosomes could be achieved by constructing a consensus map from two or more bi-parental populations.

The availability of the draft genome sequences of hexaploid wheat [47, 53] and barley [48, 54] greatly facilitated the construction of the linkage map in this work. Chromosome assignments and identification of inverted linkage groups were accomplished without anchor markers through the integration of 64 bp of GBS SNP tags into draft sequences of these species. As expected, more SNP tags were anchored to hexaploid wheat than barley. However, assignment of the linkage groups into their respective chromosomes still had high integrity with barley as there was a one-to-one relationship between *Ae. umbellulata* and barley chromosomes. The pattern of syntenic relationship observed between hexploid wheat and *Ae. umbellulata* in the current study is broadly similar with previously reported results based on wheat markers [40, 55, 56] except for chromosome 7U that shared segments with group 3 chromosomes of wheat instead of group 6. From the syntenic relationship between *Ae. umbellulata* and barley, chromosome 7U had common markers with 7H and 3H but none with 6H (Fig. 5). The relationship between 4U and group 1 of wheat is not known in previous observations. The previously reported syntenic relationships between wheat and *Ae. umbellulata* were established based on few markers per chromosome and only show macro-level synteny between the two species. However, generating large number of markers per chromosome as done in the current work may allow to access regions of chromosomes that not represented when sparse markers per chromosomes are used.

Seedling infection type data recorded after inoculating the F_2:3_ families allowed accurate resistance classification of F_2_ plants. With genome-wide QTL scanning methods, a major QTL region conferring resistance to stem rust pathogen races TTTTF and TTKSK was identified on chromosome 2U. When mapped as a qualitative trait, the linkage map position of the resistance gene also agreed with the detected QTL region. Group 2 chromosomes of hexaploid wheat also harbor 19 stem resistance QTL [57] including major genes such as *Sr32* (chrs 2A, 2B and 2D), *Sr21* (chr 2A), *Sr39, Sr36, Sr47, Sr28* and *Sr9* (chr 2B) and *Sr46* (chr 2D). However, the newly mapped QTL in the current investigation and the *Ae. umbellulata*-derived leaf rust resistance gene (*Lr9*) are located on different chromosomes as the latter was derived from chromosome 6U and the introgressed segment was also mapped on chromosome arm 6BL of hexaploid wheat [58, 59]. This demonstrates that *Ae. umbellulata* is a source of untapped rust resistance genes that need to be exploited in the future.

## Conclusions

This study presents the first QTL detected for stem rust resistance from *Ae. umbellulata*, a wild relative of wheat. The new QTL was mapped on chromosome 2U using an F_2:3_ bi-parental population with GBS markers. The stem resistance QTL-associated markers in the current study can facilitate a rapid selection of wheat*-umbellulata* recombinant events in alien gene introgression breeding programs and aid in the cloning of this gene.

## Additional files

Additional file 1: Table S1. Chromosomal locations and genotypic data for GBS markers. (XLSX 924 kb)
Additional file 2: Table S2. QTL detected by Single QTL mapping methods. (XLSX 17 kb)

## Abbreviations

cM: Centimorgan
GBS: Genotype-by-sequencing
LOD: Logarithm of odds
MQM: Multiple QTL mapping
QTL: Quantitative trait loci
RFLP: Restriction fragment polymorphism
SNP: Single nucleotide polymorphism
WGS: Whole genome sequence
